# Additive prognostic impact of the cerebrospinal fluid arginine/ornithine ratio to established clinical scores in aneurysmal subarachnoid hemorrhage

**DOI:** 10.3389/fneur.2023.1156505

**Published:** 2023-04-14

**Authors:** Johannes Weller, Tim Lampmann, Harun Asoglu, Matthias Schneider, Stefan Felix Ehrentraut, Felix Lehmann, Erdem Güresir, Franziska Dorn, Gabor C. Petzold, Hartmut Vatter, Julian Zimmermann

**Affiliations:** ^1^Department of Neurology, University Hospital Bonn, Bonn, Germany; ^2^Department of Neurosurgery, University Hospital Bonn, Bonn, Germany; ^3^Department of Anesthesiology, University Hospital Bonn, Bonn, Germany; ^4^Department of Neuroradiology, University Hospital Bonn, Bonn, Germany; ^5^German Center for Neurodegenerative Diseases (DZNE), Bonn, Germany

**Keywords:** subarachnoid hemorrhage, outcome prognostication, ornithine (PubChem CID: 6262), arginine (PubChem CID: 6322), scores accuracy, cerebrospinal fluid, arginase 1

## Abstract

Cerebrospinal fluid (CSF) metabolites are increasingly recognized as prognostic factors in aneurysmal subarachnoid hemorrhage (SAH). The CSF arginine/ornithine ratio (Arg/Orn) was shown to predict cerebral vasospasms and clinical outcome in SAH. The additive prognostic value of Arg/Orn over established prognostic scores has not been investigated. CSF Arg/Orn and the established prognostic scores SAH, FRESH, SAH-PDS, HAIR, Rosen–McDonald, Hunt and Hess, WFNS and modified Fisher scale were determined in a prospective cohort of patients with aneurysmal SAH. Logistic regression models to predict a favorable outcome, defined as a modified Rankin Scale score of 0–3 at 3  months follow-up, were constructed for each score, both with and without the addition of Arg/Orn. The impact of Arg/Orn was assessed comparing logistic regression models containing the respective score with and without Arg/Orn with the likelihood ratio chi-squared test. CSF Arg/Orn and clinical scores were determined in 38 SAH patients. Arg/Orn was an independent predictor of clinical outcome when added to established prognostic scores (*p* < 0.05) with the exception of HAIR (*p* = 0.078). All models were significantly improved if Arg/Orn was added as a covariable (*p* < 0.05). The results of this study confirm Arg/Orn as an independent prognostic factor and its addition improves established prognostic models in SAH.

## 1. Introduction

Aneurysmal subarachnoid hemorrhage (SAH) is a form of hemorrhagic stroke with high morbidity and mortality (1). A distinctive feature of SAH is the bipartite course of brain damage. Patients surviving the early phase after SAH, characterized by the transient hemorrhage from a ruptured cerebral aneurysm and its endovascular or surgical securing to prevent re-bleeding, might experience delayed neurological deficits due to delayed cerebral ischemia (DCI), which substantially contribute to poor outcome. DCI typically occurs 3–14 days after the initial bleeding, affects up to 30% of patients and may result in cerebral infarction (2, 3). Although the pathophysiology of DCI remains elusive, it is generally accepted that intracellular products released from erythrocytes to the subarachnoid space contribute to cerebral vasospasms and impaired microcirculation (1, 4). Several mechanisms have been suggested, including nitric oxide (NO) scavenging, inflammation, microvascular thrombosis, and cortical spreading depression (5–8). We have recently shown that arginase-1 is released from lysed erythrocytes to the subarachnoid space in SAH (9). Arginase-1 metabolizes L-arginine to L-ornithine and urea, thus depleting L-arginine, the educt for NO synthesis by the endothelial nitric oxide synthetase (10). The arginine/ornithine ratio (Arg/Orn) is an established marker of arginase activity in systemic diseases (11). Arg/Orn in the cerebrospinal fluid (CSF) as a marker of arginase-1 levels is decreased in SAH patients and a low ratio predicts vasospasms and poor outcome already within the first 72 h after initial bleeding (9).

Numerous systems were developed for grading the clinical condition of patients with SAH (12), and several prognostic scores have been developed to predict outcome and mortality in aneurysmal SAH (13–20). These consider differing combinations of demographic (16–19), clinical (13, 15–20), neuroimaging (14, 16, 18) and laboratory variables (13, 19) (Table 1). The aim of this study is to investigate the additive prognostic impact of Arg/Orn in the CSF to several commonly used scores for outcome prediction in aneurysmal SAH.

**Table 1:** Overview of prognostic scores and included variables.

Score	Demographic	Clinical	Imaging	Lab
SAH-PDS [Physiologic Derangement Score, (13)]		+		+
mFS [modified Fisher Scale, (14)]			+	
Hunt and Hess (15)		+		
HAIR [Hunt and Hess, Age, Intraventricular hemorrhage, Re-bleed, (16)]	+	+	+	
SAH [clinical Status, Age, Health conditions, (17)]	+	+		
Rosen and Macdonald (18)	+	+	+	
FRESH [Functional Recovery Expected after Subarachnoid hemorrhage, (19)]	+	+		+
WFNS [World Federation of Neurosurgical Societies, (20)]		+		

## 2. Materials and methods

### 2.1. Study design

This study uses data from a prospective, non-interventional clinical trial performed at the author’s tertiary university hospital between 09/2016 and 05/2019 (9). The study was carried out according to the Declaration of Helsinki and approved by the local Ethics Committee (protocol code 375/16). As described previously, patients aged 18 years or older with aneurysmal SAH with Fisher grade 3 with or without intraventricular hemorrhage and with ventricular drainage insertion as indicated by the treating clinician were included (9). Exclusion criteria were mycotic aneurysms, known chronic infections, hemophilia and missing relatives if patients were unable to consent. In the present analysis, only patients with available 90-day follow-up and available CSF within 10 days after the initial bleeding event were included. Treatment was performed according to the standardized diagnostic and therapeutic regimen at the author’s institution. All patients underwent computed tomography (CT) scan at admission followed by CT and/or digital subtraction angiography, and aneurysm treatment was based on interdisciplinary consensus. Further clinical, imaging and baseline characteristics as well as laboratory values were collected as required by the included prognostic scores (13–20).

The primary endpoint was a favorable neurological outcome, defined as a modified Rankin Scale (mRS) score of 0–3 at 90-day follow-up, obtained as patient visit.

### 2.2. Sample collection, preparation and biochemical analysis

Details on the methods as well as the time course of the individual measurement results of Arg/Orn have already been published in detail (9). CSF samples collected from the ventricular drainage between day 1 and day 10 (median day: 4, interquartile range (IQR) 2–6, range 1–10) were included in the present analysis. Samples were immediately centrifuged and stored at − 80°C. L-arginine and L-ornithine were measured by either high-performance liquid chromatography (HPLC) or liquid chromatography tandem mass spectrometry (LC–MS) in certified clinical laboratories using commercially available protocols for CSF amino acid profiling without modification. If more than one CSF sample from a patient was available, mean values were calculated (number of samples per patient: median 2, IQR 1–3, range 1–5).

### 2.3. Statistical analysis

Descriptive statistics are provided for baseline characteristics, prognostic scores and outcome parameters. Logistic regression models for prediction of a favorable neurological outcome were constructed for each score with and without the inclusion of Arg/Orn as a prognostic parameter, and odds ratios (OR) with 95% confidence intervals (CI) are reported for all predictors. The predictive power of the logistic regression models was assessed with the corrected Akaike’s Information Criterion (AIC), where lower AIC values indicate higher prognostic accuracy of the respective model. Model improvement by introduction of Arg/Orn as a prognostic parameter was formally assessed by the likelihood ratio chi-square test comparing the respective full and reduced model. Receiver operating characteristic curves were derived for the respective models. Statistical significance was accepted at an alpha level of *p* < 0.05 and analyses were two-sided. All statistical analyses were performed with R (R Core Team, 2022, version 4.2.1).

## 3. Results

### 3.1. Cohort characteristics

Subarachnoid hemorrhage patients (38) with available CSF and 90-day follow-up data were included in the analysis. 55% were female, the mean age was 59.2 years (standard deviation, 11.7), and the aneurysm was secured with clipping in 45% and endovascular coiling in 55%. The median mRS at 90-day follow-up was 4 (IQR 1–5) and 45% achieved a favorable neurological outcome. Arg/Orn was determined using HPLC in 60% of patients and LC–MS in 40%.

### 3.2. Prognostic models

Median values of the included prognostic scores are reported in Table 2 and logistic regression models were constructed for all included prognostic scores. In univariable logistic regression analysis, Arg/Orn was a significant predictor of favorable neurological outcome with an OR of 2.25 (95% CI, 1.32–4.53, *p* < 0.01) per increment. Univariable analyses confirmed all established prognostic scales but SAH PDS (*p* = 0.18) as significant predictors of a favorable neurological outcome in our cohort (*p* < 0.05; Table 2). In multivariable regression models including Arg/Orn in addition to the respective prognostic scale, the former was an independent prognostic factor (*p* < 0.05) in all cases with the exception of the HAIR scale (*p* = 0.078; Table 2).

**Table 2:** Odds ratios with 95% confidence intervals for the respective predictors derived from uni- and multi-variable regression models for the prediction of favorable neurological outcome in SAH patients.

Predictor	Median score (IQR)	ORs of reduced model	ORs of full model	
			Predictor	Arg/Orn
SAH score	4 (2.25–5)	0.67 (0.43–0.96)^*^	0.71 (0.43–1.10)	2.08 (1.22–4.21)^*^
FRESH	3.72 (1.97–6)	0.59 (0.37–0.88)^*^	0.71 (0.42–1.12)	1.87 (1.09–3.82)^*^
SAH PDS	1 (0–1.75)	1.39 (0.89–2.41)	1.78 (1.01–3.79)	2.63 (1.09–3.82)^*^
HAIR	1.5 (1–4)	0.58 (0.35–0.85)^*^	0.71 (0.42–3.79)	1.77 (1.01–3.70)
Rosen and Macdonald	7 (5.25–8)	0.64 (0.26–0.84)^*^	0.68 (0.40–1.08)	2.07 (1.20–4.22)^*^
Hunt and Hess	3 (2–5)	0.49 (0.26–0.84)^*^	0.60 (0.30–1.12)	1.91 (1.12–3.82)^*^
WFNS	4 (2–5)	0.61 (0.38–0.92)^*^	0.73 (0.44–1.18)	2.00 (1.16–4.05)^*^
Modified Fisher Scale	3 (3–4)	0.18 (0.02–0.85)^*^	0.41 (0.05–2.52)	2.02 (1.16–4.18)^*^
Arg/Orn	3.7 (2.3–4.5)	2.25 (1.32–4.53)^**^	NA	NA

^*^p < 0.05, ^**^p < 0.01.

Reduced models contain the respective score, full models contain the Arg/Orn in addition to the respective score.

**Figure 1:**
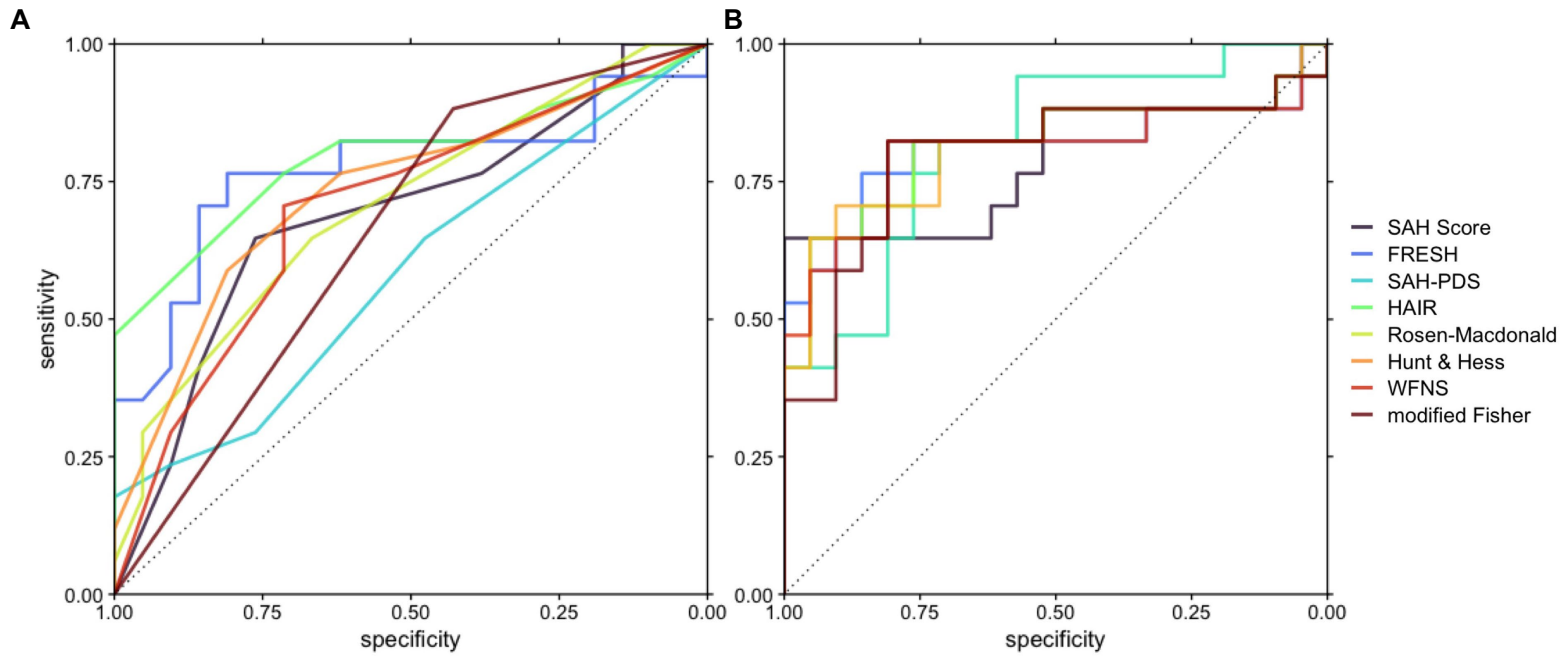
Receiver operating characteristic curves depicting the accuracy of prognostic models based on the respective scores with or without Arg/Orn. **(A)** Prognostic models including the respective score. **(B)** Prognostic models including Arg/Orn and the respective score.

All logistic regression models based on established prognostic scores were significantly improved with the introduction of Arg/Orn as a prognostic parameter (Table 3). The resulting receiver operating characteristic curves for classification of favorable neurological outcome are shown in Figure 1. The areas under the curve range from 0.5840–0.7955 for reduced models and from 0.7871–0.8179 for full models.

**Table 3:** Comparison of full versus reduced regression models.

Score	AIC		Value of *p*	Reduced model	Full model
SAH Score	51.80	46.23	0.005
FRESH	49.42	46.53	0.021
SAH PDS	54.57	44.67	<0.001
HAIR	48.11	46.49	0.046
Rosen and Macdonald	50.89	46.01	0.007
Hunt and Hess	49.68	46.11	0.015
WFNS	51.12	46.99	0.011
Modified Fisher Scale	51.87	47.77	0.011
Arg/Orn	46.31	NA	NA

A lower AIC value indicates better quality of the model. Value of *P* < 0.05 indicates that the respective full model including Arg/Orn performs better compared to the reduced model without Arg/Orn.

## 4. Discussion

SAH is a heterogeneous disease with highly variable clinical trajectories, where some patients experience devastating complications despite successful securing of the aneurysm. Based on the hypothesis that following SAH, the enzyme arginase-1 is released from lysed erythrocytes to the subarachnoid space and contributes to the depletion of the NO precursor L-arginine, we have shown that CSF Arg/Orn as an indirect measure of arginase-1 activity predicts cerebral vasospasm and clinical outcome early after SAH (9).

Early prognostication is of high clinical importance in SAH. However, it is complicated by the variable clinical course, where in particular secondary complications frequently contribute to unfavorable clinical outcomes. This led to the development of numerous prognostic scores (12). Here, we demonstrate that CSF Arg/Orn has a high prognostic significance for functional outcome after SAH and the AIC values obtained from the univariable models suggest an at least equal prognostic performance of Arg/Orn compared to established prognostic scores. Furthermore, its inclusion significantly improved all prognostic models. Of note, CSF Arg/Orn as a basic laboratory test often used in clinical routine can easily be applied and might replace or supplement the sometimes complicated prognostic scores incorporating neuroimaging, laboratory, demographic and clinical data.

In our cohort, SAH-PDS provided the lowest prognostic accuracy, which seems plausible as it captures the physiological derangement without taking SAH severity itself into account (13). Surprisingly, the combination of Arg/Orn with SAH-PDS resulted in the best combination model. Accordingly, Arg/Orn seems to best capture the severity of SAH while SAH-PDS complements the prognostication through assessment of acute physiologic derangements.

In addition to the high clinical relevance and ease of use of Arg/Orn, a key finding of our study is the description of a novel potential pathomechanism that contributes to the development of DCI in SAH. As has been shown for other hemolytic disorders such as paroxysmal nocturnal hemoglobinuria or sickle cell disease, arginase-1 is released from lysed erythrocytes and leads to vasoconstriction by L-arginine consumption (21, 22). This finding opens further therapeutic opportunities, such as blockade of arginase-1 or supplementation of arginine, to prevent DCI after SAH. Work from other groups also supports an association of arginine metabolism with the development of DCI and poorer outcome after SAB. Two independent reports using metabolomic approaches described an association between high CSF L-ornithine and poor outcome (23, 24). Further studies determining amino acid concentrations in CSF after SAH also found an association of L-ornithine and poor outcome without establishing the link to the enzyme arginase (25, 26). While these findings corroborate the observed association, our results allow for the attribution of these descriptive results to a potential pathomechanism. Taken together, the aforementioned works highlight the potential of metabolomic approaches to better understand the pathophysiology of neurovascular diseases.

Further studies should investigate the role of the glymphatic system in relation to Arg/Orn alterations following SAH. CSF and its solutes including high molecular weight proteins are transported in the glymphatic network in close spatial proximity to arterioles (27). Thereby, arginase-1 could cause local Arg/Orn alterations even remotely from the ruptured aneurysm. Delayed clearance of arginase-1 and other blood degradation products due to impaired glymphatic flow following SAH might also contribute to vasospasms and restored glymphatic clearance might normalize both local and widespread Arg/Orn changes (28). Further work should also examine the influence of Arg/Orn alterations on cortical venous filling, as a decreased cerebral venous outflow was raised as a potential marker of poor outcome in SAH (29).

This study has several limitations. In particular, the small size of the cohort and the monocentric design needs to be mentioned. Nevertheless, this prospective study was able to confirm established prognostic scores, supporting the validity of our results. Further, the inclusion of only patients with ventricular drainage placement reduces generalizability, and sample size precluded an analyses of the dependence between day of CSF sampling and prognostic relevance of Arg/Orn, although we demonstrated previously that prognostication by Arg/Orn is possible within the first 72 h after SAH (9). We could not include all established prognostic scores in this analysis because some necessary parameters, such as breathing pattern for the FOUR score, were not collected in a standardized manner (30). Other prognostic scores not included in this manuscript incorporate clinical signs or radiological findings of early brain injury, whereas Arg/Orn most likely is a predictor for secondary brain injury (31–33). A multicenter study seems warranted to confirm our hypothesis and to further evaluate the prognostic accuracy of Arg/Orn in a larger cohort, and validation in pre-clinical animal models is necessary to confirm the assumed pathophysiological relevance of our findings.

## 5. Conclusion

Arg/Orn in CSF is an independent prognostic factor in aneurysmal SAH and its evaluation improves established prognostic models, supporting the pathogenetic relevance of the enzyme arginase-1.

## Data availability statement

The original contributions presented in the study are included in the article/supplementary material, further inquiries can be directed to the corresponding author.

## Ethics statement

The studies involving human participants were reviewed and approved by Ethics Committee of the University Hospital Bonn. The patients/participants provided their written informed consent to participate in this study.

## Author contributions

JW and JZ: conceptualization and methodology, data curation, supervision and project administration. JW: formal analysis, visualization, and writing—original draft preparation. JZ: resources. JW, TL, HA, MS, SE, FL, EG, FD, GP, HV, and JZ: data acquisition, writing—review and editing. All authors have read and agreed to the published version of the manuscript.

## Funding

The publication was supported by the Open Access Publication Fund of the University Bonn.

## Conflict of interest

FD is a proctor and consultant for Balt, Cerus Endovascular, Cerenovus, Phenox; received speakers honoraria from Stryker, Acandis, Cerenovus, Asahi Inc.; and received research funding from Cerenovus.

The remaining authors declare that the research was conducted in the absence of any commercial or financial relationships that could be construed as a potential conflict of interest.

## Publisher’s note

All claims expressed in this article are solely those of the authors and do not necessarily represent those of their affiliated organizations, or those of the publisher, the editors and the reviewers. Any product that may be evaluated in this article, or claim that may be made by its manufacturer, is not guaranteed or endorsed by the publisher.
